# Effects of Glutamine and Omega-3 Fatty Acids on Erythrocyte Deformability and Oxidative Damage in Rat Model of Enterocolitis

**DOI:** 10.14740/gr683w

**Published:** 2015-10-21

**Authors:** Ruksan Cehreli, Hale Akpinar, Aysegul Temiz Artmann, Ozgul Sagol

**Affiliations:** a Department of Prevantive Oncology, Institute of Oncology, Dokuz Eylul University Inciralti, Izmir 35340, Turkey; b Division of Gastroenterology, Dokuz Eylul University School of Medicine, Inciralti, Izmir 35340, Turkey; c Department of Cell Biophysics and Cellular Engineering, Institute for Bioengineering, Aachen University of Applied Sciences, Germany; d Department of Pathology, Dokuz Eylul University School of Medicine, Inciralti, Izmir 35340, Turkey

**Keywords:** Erythrocyte deformability, Oxidative stress, Inflammatory bowel disease, Enterocolitis, Glutamine, Omega-3 fatty acids

## Abstract

**Background:**

The aim of the study was to investigate preventive effects of glutamine (Gln), omega-3 fatty acids (FA) on erythrocyte deformability (EDEF) in rat model of indomethacin-induced enterocolitis.

**Methods:**

Nineteen Wistar albino male rats were divided into three groups: control group, colitis induced by indomethacin and were fed with a standard laboratory diet (group 1), and colitis induced by indomethacin and were also fed with Gln, omega-3 FA (group 2). An investigation was performed in a rat model of experimental colitis induced by subcutaneous injections of 2 mL intdomethacine solution applied at 24 and 48 hours intervals to male Wistar rats for 14 days. Gln and omega-3 FA were added to the daily standard diets of the animals during 14 days of injections. During the study, changes in body weight were evaluated. The intestines were examined, and colitis was macroscopic and histologically scored. The circulating tumor necrosis factor alpha (TNF-α) and interleukine-1β (IL-1β), erythrocyte transit time (ETT) and thiobarbituric acid reactive substances (TBARS) levels were determined in addition to calculation of EDEF indices in all groups.

**Results:**

No significant differences in body weight changes could be determined between the standard diet and special diet groups at the end of the experiment. After macroscopic and microscopic scoring, in all of the groups that colitis was found induced, the lowest microscopic score was observed in the group 2. But Gln and omega-3 FA supplemented diet did not change the mean macroscopic and histological scores in all rats. The proliferating cell nuclear antigen (PCNA) levels were significantly higher in group 1 and group 2 compared to the control group. Effects of the diet on circulating TNF-α and IL-1β levels were found correlated with inflammation but statistically significant differences were not found in the group 1 and group 2 (P < 0.05). The ETT and TBARS levels in standard and special diet groups were significantly increased (P < 0.05). However, EDEF indices which are an important parameter of the study were decreased in indomethacin-induced enterocolitis groups that fed with standard and special diet.

**Conclusions:**

Increases in ETT and TBARS levels did not return to normal by addition of Gln and omega-3 FA to diet. Our results suggest that determination of effective optimal doses and route of administration for these nutrients may play an important role in reducing EDEF and microvascular changes.

## Introduction

Inflammatory bowel disease (IBD) occurs as a result of the combined effects of environmental changes, multiple genetic variations, oxidative stress, alterations in intestinal microflora and abberrations in innate and adaptive immune responses [1]. Local inflammatory mediators such as eicosanoids, pro-inflammatory cytokines and reactive oxygen species (ROS) play an important role in the pathogenesis of the disease [2-5]. The impairment of the gut barrier function is also the common characteristic of IBD. It may be due to changes of bacterial microflora, excessive immune cell mediated response and immune cell infiltration in the intestinal wall. These events would result in intestinal tissue destruction and the developement of IBD due to increased ROS and cytokine production [6].

Also ROS attacks may also cause membrane protein oxidation and lipid peroxidation. Erythrocyte membranes are vulnerable to lipid peroxidation because of the lipid components of their membranes.

Lipid peroxidation has adverse effects on the deformability of erythrocytes [7].

Deceleration in microvascular flow is another important factor in the etiopathogenesis of IBD which may associate with distorted erythrocyte deformability (EDEF) [8]. Although EDEF has been an intensively studied in chronic diseases, but only one clinical IBD study has been reported in the literature revealing that increased erythrocyte malonyldialdehyde content causes a reduction in EDEF [9]. In IBD microvascular flow slowdown can be expected to induce decrease in EDEF, but data on EDEF are insufficient.

Anti-inflammatory therapies are beneficial against active IBD. There is also evidence that a number of nutrients may act to suppress inflammation [10].

In recent years, nutrition has emerged as an important part of IBD therapy.

Glutamine (Gln) is a multifunctional amino acid and is also the main fuel for rapidly dividing cells such as enterocytes, colonocytes, immune cells such as lymphocytes (T and B cells) and macrophages [11-14]. Gln has also been considered as a candidate for barrier functions and antioxidant therapy in laboratory models [6].

Omega-3 fatty acids (FA) are precursors of potent lipid mediators, termed eicosanoids, and they have anti-inflammatory properties. Omega-3 FA have also been shown to alter the production of inflammatory cytokines [15] and have local and systemic supressive effects on cell mediated immunity via cytokine release (including tumor necrosis factor alpha (TNF-α)) [16].

The aim of this study was to evaluate the influence of a Gln and omega-3 FA supplemented diet on inflammation, EDEF, erythrocyte oxidative damage, and serum TNF-α and interleukine-1β (IL-1β) levels in rat model of indomethacin-induced enterocolitis.

## Materials and Methods

### Animals model

Care and handling of the animals were in compliance with the internationally accepted standard guidelines for use of animals, and approval was obtained from the animal ethics council of Dokuz Eylul University School of Medicine (DEUSM).

DEUSM Experimental Research Laboratory provided 19 Wistar albino male rats weighing 200 - 300 g used in this study. The rats were housed in standard single cages in a room under constant temperature 22 ± 2 °C, 55±5% humidity, and a 12 h light/dark cycle at the DEUSM Experimental Animal Laboratory.

Experimental animals were divided into three groups.

The control group included five rats that were not treated with indomethacin and each of the other two groups that were treated with indomethacin included seven rats.

All animals were given standard and specific diets according to the experimental design (lab diet; Oriental Yeast Company, Tokyo, Japan) and water *ad libitum*.

All rats were fasted for 16 h, and weight gains were registered before the procedure of laparotomy.

### Induction of colitis

All rats were put in a special cage where they could fit comfortably. Enterocolitis (intestinal inflammation) was induced by indomethacin at a dose of 7.5 mg/kg dissolved in 5% sodium bicarbonate given in two subcutaneous injections at 24 h interval (n = 7). The 0.5 mL physiological saline was subcutaneously administered to the rats in the control group (n = 5).

This represents a rat model for Crohn’s disease [16]. After indomethacin administration, no rats developed perforation due to the formation of ulcerations in the colon. However, indomethacin-induced gastrointestinal bleeding in rats were not counted because of exclusion.

### Experimental design

Nineteen Wistar albino male rats were divided into three groups .The rats in the control group (n = 5) were not treated with indomethacin and were fed on standard laboratory diet (lab diet; Oriental Yeast Company, Tokyo, Japan).

Group 1 (n = 7): the rats were fed with a standard laboratory diet (lab diet; Oriental Yeast Company, Tokyo, Japan), and group 2 (n = 7): in addition to standard diet, the rats were also fed with a special diet supplemented Gln (12.5% mg), omega-3 FA (8.5% g) (Oriental Yeast Company, Tokyo, Japan).

The rats received standard chow diet or a special diet for 14 days between the day before the first indomethacin injection and the fasting period before the laparatomy procedure. Their body weights were recorded daily.

### Histopathologic examination

After 14 days, 24 h of fasting blood samples were drawn from the abdominal aorta under ether anesthesia, and then the rats were sacrificed because of hypovolemia. Decapitation was performed after tissue samples were collected for pathological examination. The abdominal cavity was opened by a midline incision. The entire length of small bowel and colon, from the pylorus to the rectum was then removed. The lumen was gently flushed with phosphate-buffered saline (0.9% NaCl) for the clearance of fecal material. The lumen was then opened longitudinally, and fresh specimen was examined under a microscope at a magnification of × 5 by a pathologist who was blind to the experiment. The extent of mucosal damage was assessed by using the macroscopic inflammatory scoring (MIS) system of Wallace et al [17]. Following evaluation of macroscopic lesions, tissue sections were obtained containing the areas of gross ulcerative lesions, including the normal mucosa next to the lesions.

The tissues were fixed in formaldehyde, embedded in paraffin, and tissue sections stained with hematoxylin and eosin. Histopathologic evaluations were made under a light microscope by the same experienced pathologist who was again blind to the experiment. The lesions were scored as described by Vilaseca et al [18].

A standard streptavidin biotin immune-peroxidase method was used for proliferating cell nuclear antigen (PCNA) immunostaining (dilution: 1:100). The tissue sections were deparaffinized and rehydrated, and endogenous peroxidase activity was blocked using a 0.3% solution of hydrogen peroxide in phosphate buffered saline (PBS) at room temperature for 10 min. The sections were then boiled in citrate buffer solution in a microwave oven, three times for ten min for epitope retrieval. Primary antibodies were applied for 30 min at room temperature, and the slides were washed in TRIS buffer. Linking antibody and streptavidin peroxidase complex (LSAB kit) were added consecutively for 10 min at room temperature again followed by washing in TRIS buffer. Appropriate tissue sections were simultaneously stained with the primary antibody as positive controls. The most representative areas of the section, including mucosa next to the ulcers, were selected and marked for analysis. Antibody staining results were scored semiquantitatively.

### Serum TNF-α and IL-1β levels

Serum TNF-α and IL-1β levels were measured using a sensitive commercially available rat specific enzyme-linked immunosorbent assay (ELISA) kit following the manufacturer’s instructions (Endogen Inc., Woburn, MA, USA).

### Evaluation of erythrocyte thiobarbituric acid reactive substances (TBARS) and ETT

EDEF is the average duration of the passage of 1,000 erythrocytes through 5 µm diameter pores in milliseconds assessed using a cell transit analyzer (CTA). Venous blood samples anticoagulated with heparin were analyzed to assess the EDEF within a 30 min sampling time using a CTA. Breifly, a 20% suspension of washed erythrocytes was passed through nucleopore polycarbonate membranes with a 15 mm diameter and one 5 μm diameter pore. An increased cell transit time is reflected by a decreased EDEF [19-22].

Lipid peroxides were estimated using the method of Stock, Dormandy and Jain which measures TBARS [23, 24].

### Statistical analyses

All data are expressed as the mean ± SD (SEM). Comparisons between groups of non-parametric data were performed using the Mann-Whitney U test. Statistical analysis was performed using SPSS 15.0 software. Statistical significance was designated for P value < 0.05.

## Results

The rats in the control group gained an average of 13.1 g in weight, and the rats in the standard diet and special diet groups lost 3.4 and 4.2 g, respectively. No significant differences in body weight changes could be determined between group 1 and group 2 at the end of the experiment (Table 1).

**Table 1:** Changes in Body Weight in the Experimental Groups

Groups	Baseline weight	Final weight	Difference in body weight
Control (n = 5)	235.8 ± 19.3	248.9 ±17.9	13.1
Standard diet (n = 7)	244.5 ± 8.7	174.3 ± 6.8	-3.4
Special diet (supplemented with Gln + omega-3 FA) (n = 7)	270 ± 12.1	266.2 ± 10.3	-4.2

The results are shown as the mean ± SD.

### Histopathologic findings of intestine

Administration of indomethacin resulted in clear damage in the ileum and notably also in the proximal part of the colon. By 14 days after indomethacin administration, distinct areas of ulceration and inflammation separated by regions of grossly normal mucosa were detectable.

**Figure 1:**
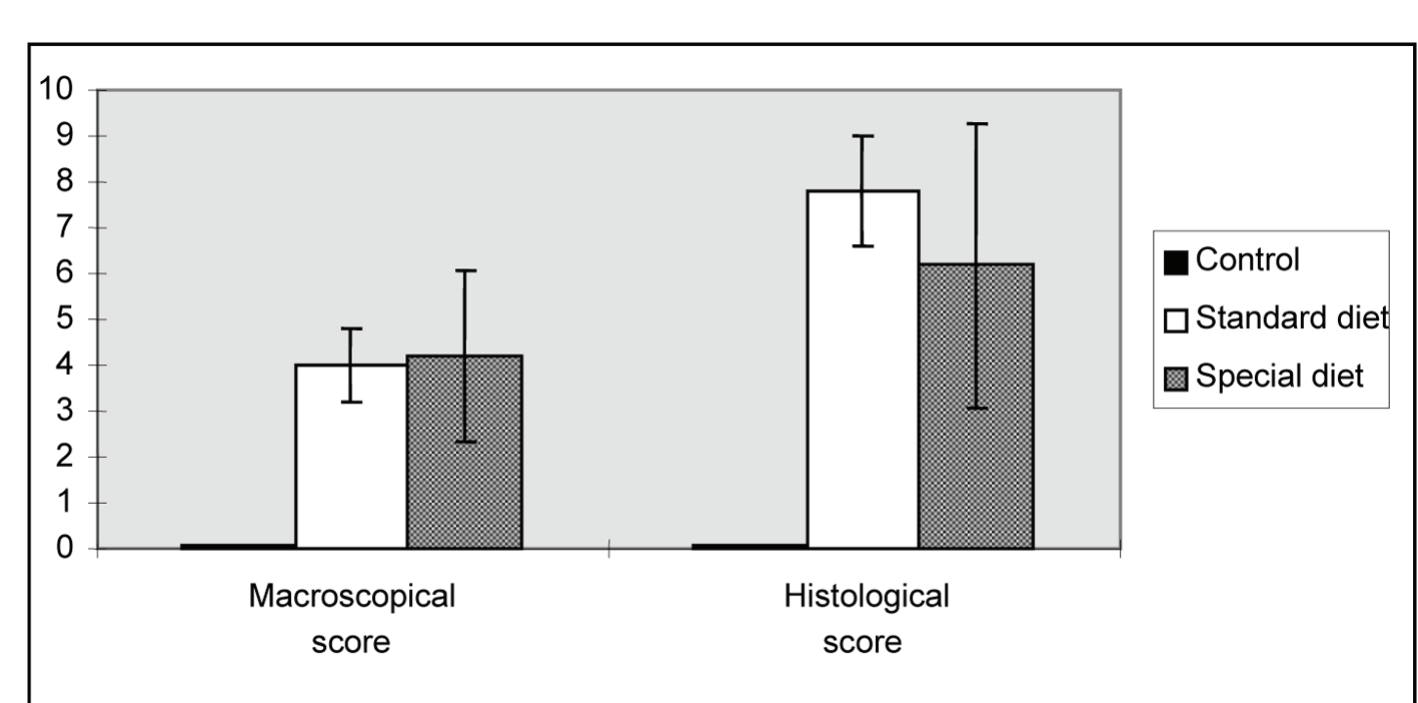
Morphological lesion scores for the intestinal damage induced by indomethacin. The results are shown as the mean ± SD.

Macroscopic ulceration was not observed in the control group. Upon macroscopic examination, a 15 mm ulcer in the intestinal tissue was observed in one rat in the standard diet group ,while three and four larger ulcers (> 2 cm) were observed in group 1 and group 2, respectively. After macroscopic and microscopic scoring, in all of the groups that colitis was found induced, the lowest microscopic score was observed in the group 2. Although these scores were significantly higher in rats fed on standard and special diets compared to rats in the control group. The standard and special diets did not alter the mean macroscopic and histological scores (Fig. 1).

**Figure 2:**
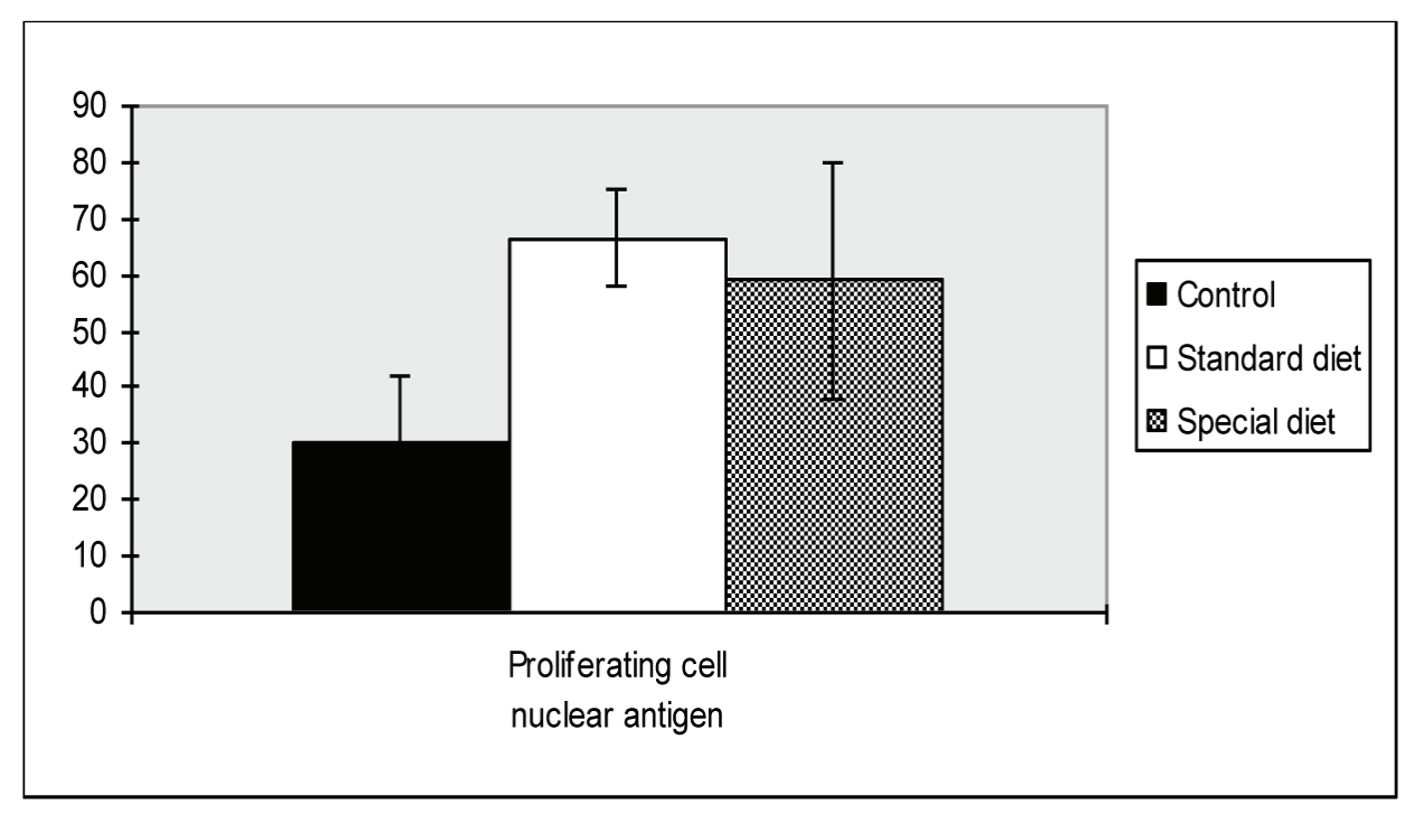
Proliferating cell nuclear antigen scores of intestine. Mean values are shown. *P < 0.05 compared to the control group.

As shown in Figure 2, PCNA levels were significantly higher in group 1 and group 2 compared to the control group (66.6 ± 23.4, 59.0 ± 21.1 vs. 30.4 ± 11.8, P = 0.018, P = 0.047, respectively).

There were no statistically differences found between group 1 and group 2.

**Figure 3:**
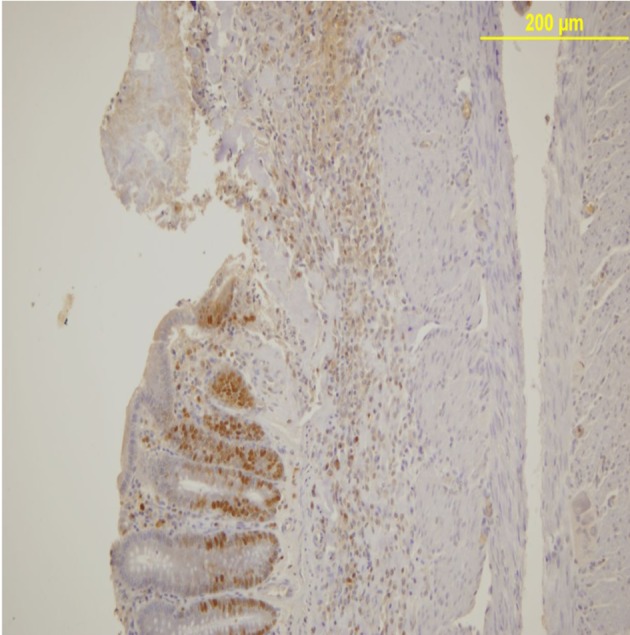
PCNA expression in the mucosal crypts adjacent to the ulcer. Immunoperoxidase staining. Original magnification, × 100.

**Figure 4:**
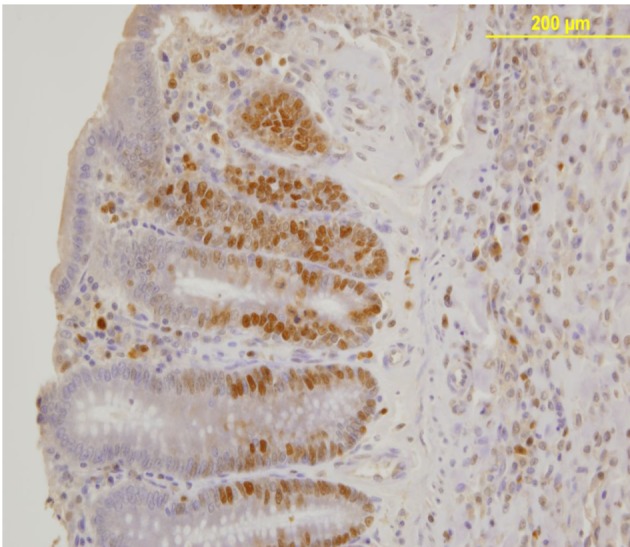
Nuclear PCNA expression in epithelial cells of the mucosal crypts. Immunoperoxidase staining. Original magnification, × 200.

PCNA expression analysis, detected by immunoperoxidase, in the epithelial cells of the mucosal crypts is shown in Figures 3 and 4.

### Serum cytokines results

**Figure 5:**
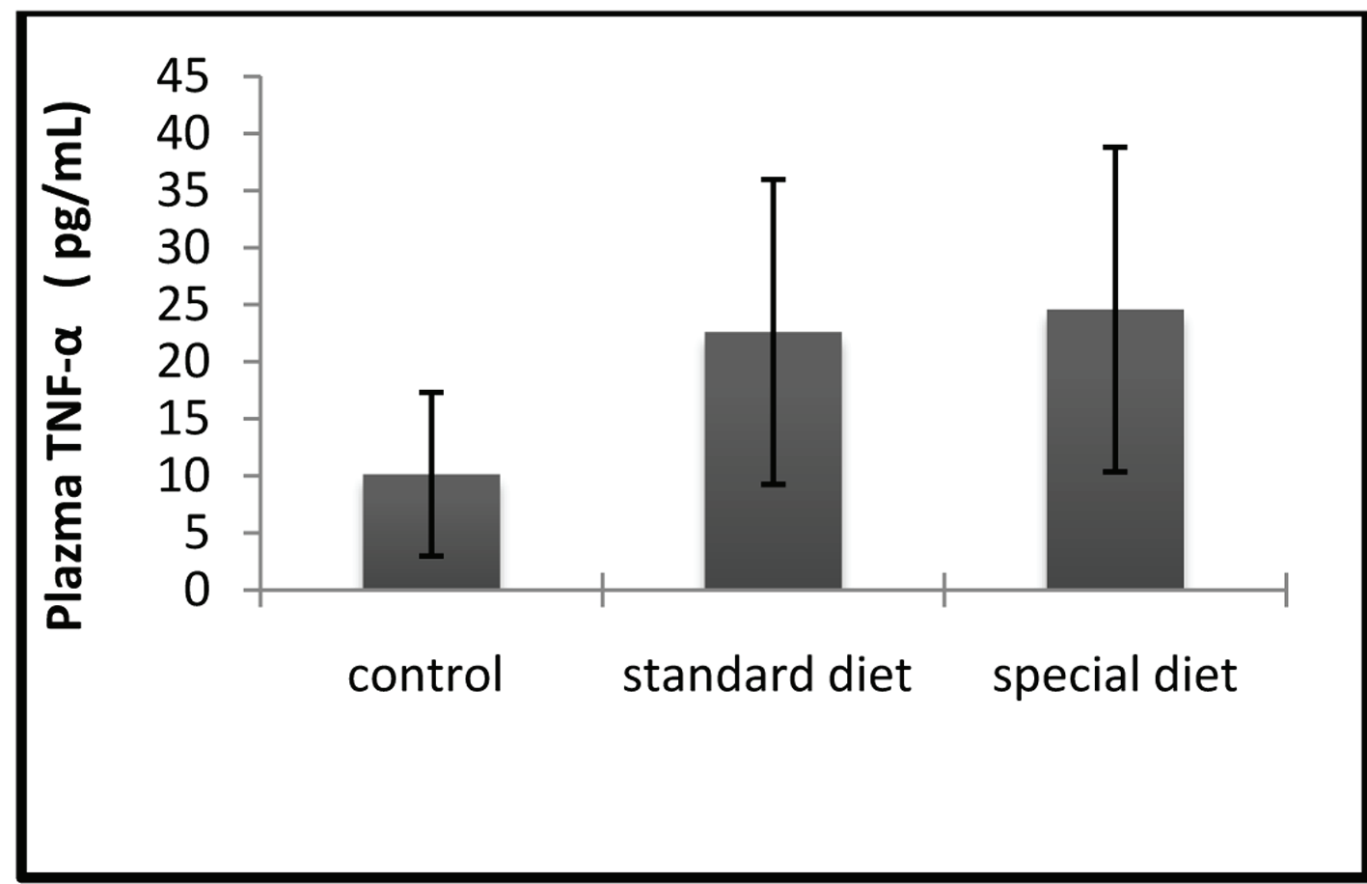
The serum TNF-α levels were comparable between groups. The results are shown as mean ± SD.

Correlation between the effects of group 2 on inflammation and increases in circulating leves of TNF-α and IL-1β, were also determined. The mean serum TNF-α level was 10.15 ± 7.16 pg/mL in the control group, 22.61 ± 13.36 pg/mL in group 1, and 24.58 ± 14.23 pg/mL in group 2 (Fig. 5).

**Figure 6:**
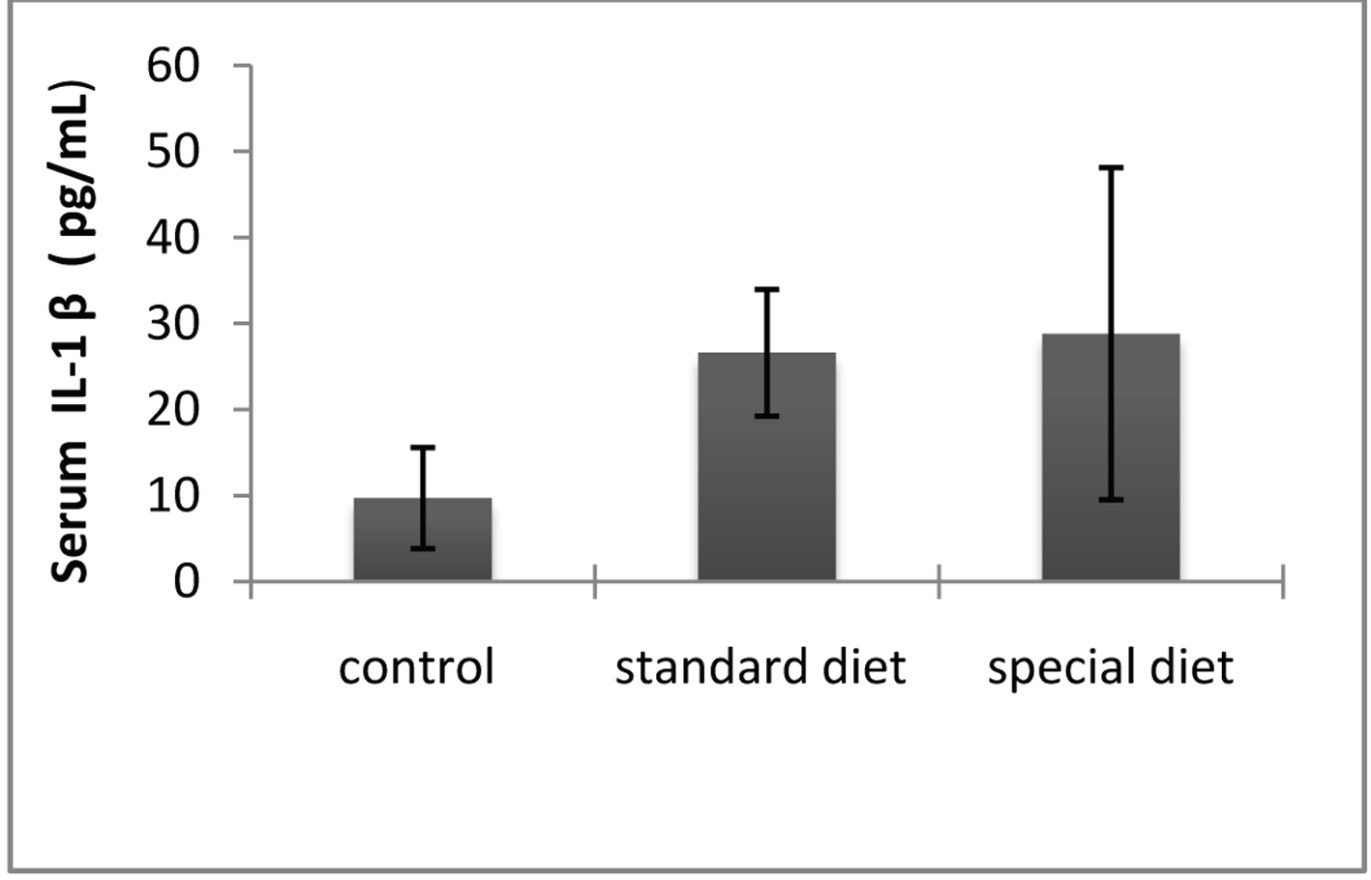
The serum IL-1β levels were comparable between groups. The results are shown as mean ± SD.

The mean serum IL-1β level was 9.71 ± 5.86 pg/mL in the control group, 25.61 ± 7.36 pg/mL in group 1, and 28.82 ± 9.31 pg/mL in group 2 (Fig. 6).

Statistically significant differences were not found in the treatment groups.

### EDEF and TBARS

**Figure 7:**
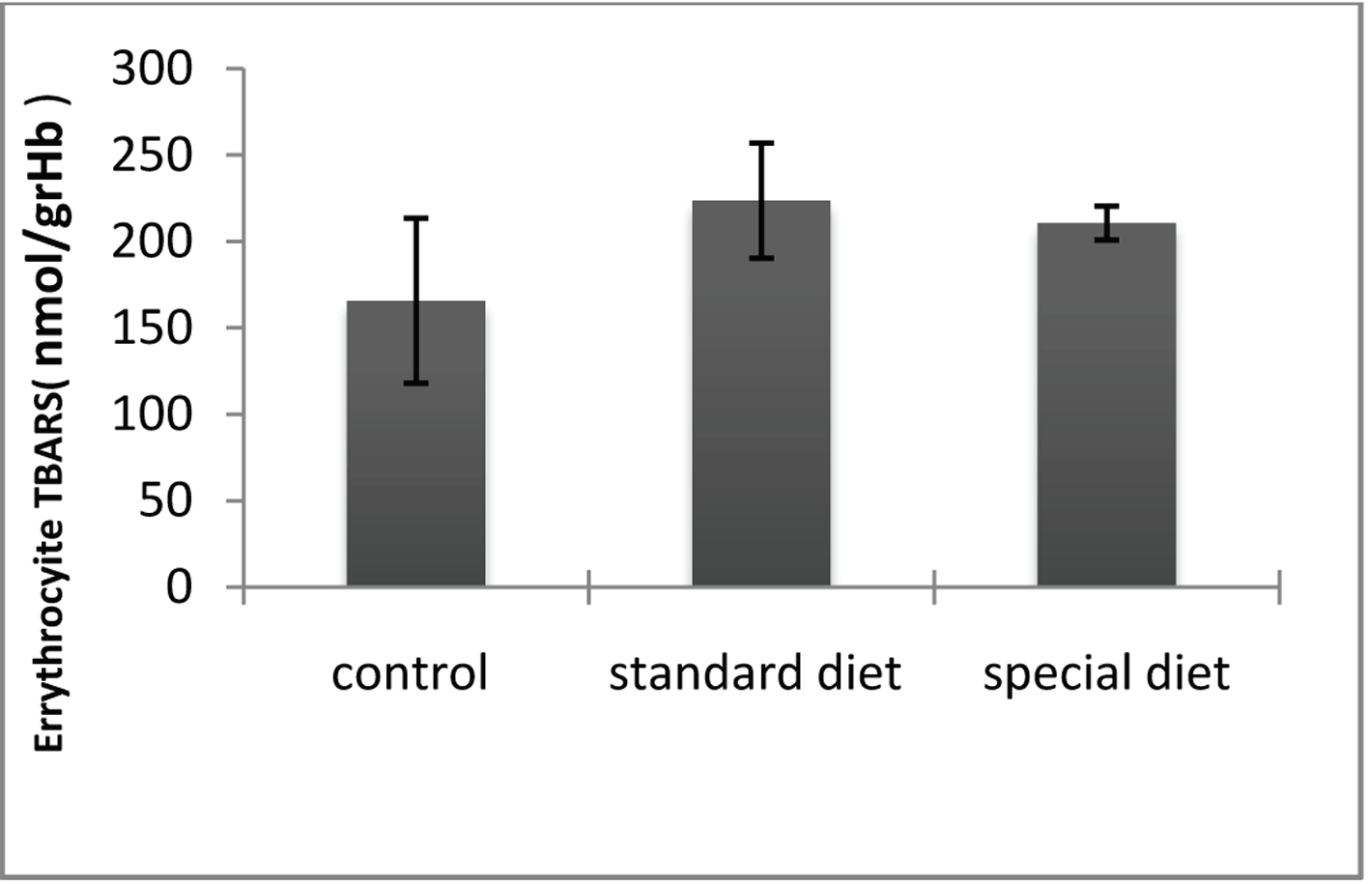
TBARS levels in erythrocytes. *P < 0.05 compared with the control group.

**Figure 8:**
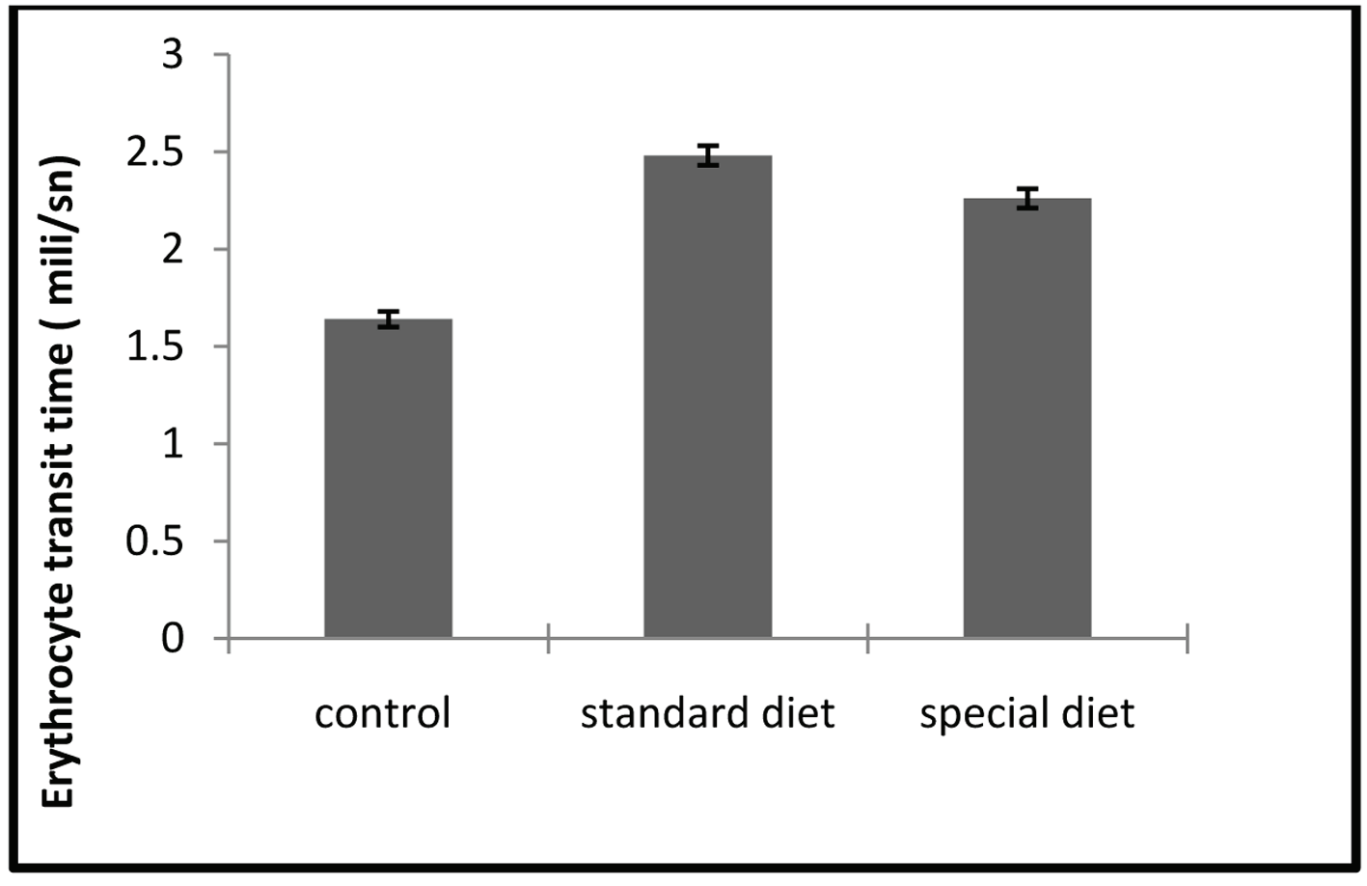
ETT assays. Each bar represents mean ± SD. *P < 0.05 compared to control group.

The ETT and TBARS levels in standard and special diet groups were significantly increased (P < 0.05; Fig. 7, 8).

The mean plazma TBARS level was 165.65 ± 47.76 nmol/g Hb in the control group, 223.61 ± 33.36 nmol/g Hb in group 1 and 210.58 ± 9.72 in nmol/g Hb in group 2. Although the intestinal inflammation caused significant increases in the TBARS levels compared to control group, they did not return to control levels when the animals were placed on the special diet (Fig. 7).

The mean ETT level was 1.64 ± 0.04 ms in the control group, 2.48 ± 0.05 ms in group 1 and 2.26 ± 0.05 ms in group 2.

Although intestinal inflammation caused a significant increase in ETT compared to control group, this did not return to the control level when the animals were applied a special diet (Fig. 8).

## Discussion

There have been a fairly small number of studies directed toward the therapeutic use of Gln and omega-3 FA for the treatment of IBD. However very limited number of preclinical studies consistently supported a positive role of Gln as a therapeutic modality in IBD. In various models of chemicals-induced colitis,treatments with enteral or local Gln and omega-3 FA were shown to improve a variety of clinical, cellular, pathohistological and biochemical endpoints of colitis.[4, 25].

Macroscopic and microscopic damage [25-27], enhanced epithelial apoptosis [28], impaired gut barrier function [29] reduced tissue antioxidant defense/enhanced oxidative stress [30-33] in addition to expression of proinflammatory mediators [25, 34].

There is only one clinical study showing the relationship between EDEF and pathophysiology of IBD in the literature [9]. However, data showing the effect of Gln and omega-3 FA on the relation between EDEF and IBD, could not be found.

In animals, it has been shown that Gln protects the intestinal mucosa in different models of IBD [29, 35]. The beneficial role of Gln in these experimental models of enterocolitis can be explained through different mechanisms [30], and it has potent antioxidant properties as well [36-38].

Antioxidant and anti-inflammatory effects induced by combination of omega-3 FA and Gln were expected to affect these mechanisms in our study, but the results of histopathological findings, and assessments of the levels of serum cytokines, erythrocyte TBARS and EDEF failed to reveal any beneficial effects of special diet.

Crohn’s disease often affects the distal ileum and colon. Gln is the preferred fuel of enterocytes whereas butyrate is preferred by the colonocytes. Gln is absorbed in the proximal part of the small intestine and therefore, cannot reach to the inflamed part of the intestine [30]..But our knowledge in this regard is insufficient.

The route of administration and dose levels are also very important for the use of Gln since as excess amounts were found to have deleterious effects on ulcers in an experimental colitis model [34, 35]. Because Shinozaki et al reported that excess Gln exacerbates toxic colitis in rats [38].

In our study, physiological dose and enteral route were used to induce a synergistic

effects of Gln and omega-3 FA, but we did not observe any beneficial effects on macroscopic and histological scores. Also omega-3 FA promotes the production of less inflammatory eicosanoids from EPA and have other anti-inflammatory actions including decreases in leukocyte chemotaxis, adhesion molecule expression, T cell reactivity and inflammatory cytokine productions [39, 40].

The reduction of chemically induced colonic inflammation and damage by omega-3 FA has been demonstrated in numerous experimental animal models [19, 41-44]. Furthermore, there is strong indirect evidence supporting the potential of omega-3 FA in modulating intestinal inflammation [44].

Although we did not observe any beneficial effects on cytokine levels of (TNF-α, IL-1β), TBARS, macroscopic and histological scores with Gln and omega-3 FA in our studies. To determine the effectiveness of omega-3 FA in an animal model, we adopted an indomethacin-induced enterocolitis rat model in our present study. Ineffectiveness of omega-3 FA observed in our experiments may be due to use of a selected chemical to induce enterocolitis and the combined use of omega-3 FA with Gln which has been shown to have some deleterious effects in excess doses [38].

In our study, scoring of the small intestine was also performed, but in a similar study scoring of the colon should be included into the study, because IBD may involve both intestine and colon as seen in Crohn’s disease.

Further aim of our study was to evaluate the *in vivo* IBD effects on EDEF and the oxidative damage marker TBARS.

Microvascular flow deceleration may play an important role in the etiopathogenesis of IBD. There are some findings that hypoperfusion and ischemia in the intestinal tissue of IBD patients are related to microvascular dysfunction [7]. The severity of intestinal injury has also been correlated with the degree of vascular injury [45-47]. Generated ROS are related to chronic inflammation that results in NF-kB activation. This also induces prolonged neutrophil infiltration and microvascular dysfunction and impairment of tissue oxygenation by increasing erythrocyte aggregation.

Elevated blood and plasma viscosity as well as impaired EDEF may produce retardation and stoppage of the flow of erythrocytes in microvessels.

Moreover, intensified erythrocyte aggregation resulting in subsequent local accumulation has

been shown to be a key factor in microcirculation disruption [48]. There are also some studies reporting that ROS increase decrease in EDEF [8].

Only one clinical study of IBD has been reported to date. Akman et al showed that increased erythrocyte malonyldialdehyde values cause a reduction in EDEF [9]. It has been shown that increased TBARS levels can decrease EDEF in our study. In addition, we suggested that anti-inflammatory and antioxidant natures of Gln and omega-3 FA would exert protective effects on EDEF and TBARS levels, but we did not observe any statistically significant changes in these parameters in group 1 and group 2.

We believe that inadequate nutrient dose and duration of treatment may be not effective in our treatment groups. However, we were also unable to assess our results in the context of other reports, because there has been no previous study of EDEF in a experimental enterocolitis model.

Further studies will thus be required to clarify this issue. There is a need to specifically monitorize effects of nutrients on plasma erythrocytes.

In conclusion, we did not observe the expected synergistic effects antioxidant and anti-inflammatory effects of a combined use of Gln and omega-3 FA on EDEF in an indomethacin-induced enterocolitis rat model. Various causes may account for the observed disappointment results.

Initial, the doses of omega-3 FA and Gln used might be too low (or nonpharmacologic) to reveal a clinical therapeutic effect. Consistently, positive results obtained in the study of critical illness have used higher “pharmacological” Gln and omega-3 FA doses that exceed the usual physiological requirements, but it would be difficult to identify which is the optimal dose of omega-3 FA and Gln.

Finally, further studies are required for the determination of optimal route of administration and doses for Gln and omega-3FA and to clarify EDEF, erythrocyte aggregation and microvascular changes in inflammation.
